# Supplementary material for: Resolving the full spectrum of human genome variation using Linked-Reads
Source: Genome Res. 2019 Apr;29(4):635–45. doi: 10.1101/gr.234443.118 (PMC6442396; doi:10.1101/gr.234443.118)
Supplement: Supplemental Material [file supp_gr.234443.118_Supplemental_longranger-2.2.zip › longranger-2.2.tar.bz2!/lr2.2-public/lib/stan/cmdstan/stan/doc/api/html/prob__grad_8hpp_source.html]

Stan: src/stan/model/prob\_grad.hpp Source File


|  |  |
| --- | --- |
|  | Stan  2.5.0  probability, sampling & optimization |


- Main Page
- Related Pages
- Namespaces
- Classes
- Files

- File List
- File Members

All Classes Namespaces Files Functions Variables Typedefs Enumerations Enumerator Friends Macros Pages

- src
- stan
- model

prob\_grad.hpp

Go to the documentation of this file.

1 #ifndef STAN\_\_MODEL\_\_PROB\_GRAD\_HPP

2 #define STAN\_\_MODEL\_\_PROB\_GRAD\_HPP

3

4 #include <utility>

5 #include <vector>

6

7 #include <stan/io/csv\_writer.hpp>

8

9 namespace stan {

10

11  namespace model {

12

18  class prob\_grad {

19

20  protected:

21

22  size\_t num\_params\_r\_\_;

23  std::vector<std::pair<int,int> > param\_ranges\_i\_\_;

24

25  public:

26

27  prob\_grad(size\_t num\_params\_r)

28  : num\_params\_r\_\_(num\_params\_r),

29  param\_ranges\_i\_\_(std::vector<std::pair<int,int> >(0)) {

30  }

31

32  prob\_grad(size\_t num\_params\_r,

33  std::vector<std::pair<int,int> >& param\_ranges\_i)

34  : num\_params\_r\_\_(num\_params\_r),

35  param\_ranges\_i\_\_(param\_ranges\_i) {

36  }

37

38  virtual ~prob\_grad() { }

39

40  inline size\_t num\_params\_r() const {

41  return num\_params\_r\_\_;

42  }

43

44  inline size\_t num\_params\_i() const {

45  return param\_ranges\_i\_\_.size();

46  }

47

48  inline std::pair<int,int> param\_range\_i(size\_t idx) const {

49  return param\_ranges\_i\_\_[idx];

50  }

51

52  };

53  }

54 }

55

56 #endif

stan::model::prob\_grad

The prob\_grad class represents the basic parameter holders for a model.

**Definition:** prob\_grad.hpp:18

stan::model::prob\_grad::num\_params\_r\_\_

size\_t num\_params\_r\_\_

**Definition:** prob\_grad.hpp:22

stan::model::prob\_grad::prob\_grad

prob\_grad(size\_t num\_params\_r)

**Definition:** prob\_grad.hpp:27

stan::model::prob\_grad::param\_range\_i

std::pair< int, int > param\_range\_i(size\_t idx) const

**Definition:** prob\_grad.hpp:48

stan::model::prob\_grad::param\_ranges\_i\_\_

std::vector< std::pair< int, int > > param\_ranges\_i\_\_

**Definition:** prob\_grad.hpp:23

stan::model::prob\_grad::~prob\_grad

virtual ~prob\_grad()

**Definition:** prob\_grad.hpp:38

stan::model::prob\_grad::prob\_grad

prob\_grad(size\_t num\_params\_r, std::vector< std::pair< int, int > > &param\_ranges\_i)

**Definition:** prob\_grad.hpp:32

stan::model::prob\_grad::num\_params\_r

size\_t num\_params\_r() const

**Definition:** prob\_grad.hpp:40

csv\_writer.hpp

stan::model::prob\_grad::num\_params\_i

size\_t num\_params\_i() const

**Definition:** prob\_grad.hpp:44


---

[ Stan Home Page ]

*© 2011–2014,
Stan Development Team.*
